# Multi-Step Regulation of the TLR4 Pathway by the miR-125a~99b~let-7e Cluster

**DOI:** 10.3389/fimmu.2018.02037

**Published:** 2018-09-07

**Authors:** Graziella Curtale, Tiziana A. Renzi, Massimiliano Mirolo, Lorenzo Drufuca, Manuel Albanese, Mariacristina De Luca, Marzia Rossato, Flavia Bazzoni, Massimo Locati

**Affiliations:** ^1^Department of Medical Biotechnologies and Translational Medicine, University of Milan, Milan, Italy; ^2^Humanitas Clinical and Research Center, Rozzano, Italy; ^3^Department of Medicine, Division of General Pathology, University of Verona, Verona, Italy

**Keywords:** innate immunity, macrophage, TLR, miRNA, IL-10

## Abstract

An appropriate immune response requires a tight balance between pro- and anti-inflammatory mechanisms. IL-10 is induced at late time-points during acute inflammatory conditions triggered by TLR-dependent recognition of infectious agents and is involved in setting this balance, operating as a negative regulator of the TLR-dependent signaling pathway. We identified miR-125a~99b~let-7e as an evolutionary conserved microRNA cluster late-induced in human monocytes exposed to the TLR4 agonist LPS as an effect of this IL-10-dependent regulatory loop. We demonstrated that microRNAs generated by this cluster perform a pervasive regulation of the TLR signaling pathway by direct targeting receptors (TLR4, CD14), signaling molecules (IRAK1), and effector cytokines (TNFα, IL-6, CCL3, CCL7, CXCL8). Modulation of miR-125a~99b~let-7e cluster influenced the production of proinflammatory cytokines in response to LPS and the IL-10-mediated tolerance to LPS, thus identifying this gene as a previously unrecognized major regulatory element of the inflammatory response and endotoxin tolerance.

## Introduction

The innate immune system detects infections via the recognition of various pathogen-associated molecules by means of specific families of pattern recognition receptors, including Toll-like receptors (TLR). TLR triggers signaling cascades leading to the production of pro-inflammatory cytokines, which promote inflammation at the site of infection and coordinate the immune response. To efficiently induce protection from pathogens, the inflammatory response must be balanced by inhibitory pathways promoting its own resolution and allowing tissue healing and regeneration. For its well documented anti-inflammatory role, mainly based on the ability to inhibit production of pro-inflammatory cytokines and simultaneously increase anti-inflammatory molecules, Interleukin 10 (IL-10) is a fundamental element of such balance. This anti-inflammatory mediator, which is produced with delayed kinetics after LPS recognition, operates as a key mediator of resolution of the acute inflammatory response and is involved in the LPS desensitization process, also known as endotoxin tolerance (1–3). Conversely, in case of pathogen persistence lymphocyte-derived signals contribute to establish a chronic immune response. Among these, a major role is played by the Th1-derived IFNγ, which counteracts IL-10 biological activities and reverses (4) the endotoxin tolerance state.

MicroRNA (miR) are endogenous small non-coding RNA molecules which function as post-transcriptional regulators of gene expression by recognizing short complementary miR-responsive elements (MRE) within the 3′-untranslated region (3′UTR) of target mRNA (5). MiR are involved in several biological processes, including the fine tuning of innate immune responses (6). TLR triggering in particular set in motion a regulatory network supported by a defined set of miR which finely tunes the TLR signaling pathway (7–15). MiR are also involved in the resolution phase of inflammation, as reported for miR-466l which promotes inflammation when early expressed in polymorphonuclear neutrophils and contributes to its resolution when expressed at later time points in macrophages engaged by apoptotic neutrophils (16). In this setting, miR activity is tightly connected with other anti-inflammatory mediators, as exemplified by the inhibitory effect of IL-10 on the expression of the proinflammatory miR-155 (17) and, on the other hand, by the recent identification of some miR, including miR-146b and miR-187, which are directly induced by IL-10 and contributes to its anti-inflammatory activities, thus acting as intracellular mediators of this and other pro-resolving mediators (14, 18, 19). Similarly, we recently reported that the glucocorticoid-responsive miR-511-5p controls the TLR4 signaling pathway (20). This study further contributes to the molecular definition of this complex network describing the anti-inflammatory miR-125a~99b~let-7e cluster, which is late-induced by TLR agonists via the IL-10-dependent regulatory loop and operates a pervasive negative regulation on the TLR signaling pathway controlling a particularly wide set of target genes involved in this pathway. We show the biological relevance of this miR-mediated regulatoy network by providing evidence of its involvement in the IL-10-mediated tolerance to LPS, thus candidating this miR cluster as a key feedback modulator of the inflammatory response.

## Materials and methods

### Reagents

LPS from *E. coli* (serotype 055:B5), palmitoyl-3-cysteine-serine-lysine-4 (pam3CSK4), imiquimod, CpG DNA oligonucleotide, and poly (I:C) were from Enzo Life Sciences. IL-10 and TGFβ were from R&D System. IFNγ and IL-1β were from Peprotech. AG-490 was from Calbiochem. Antibodies for ChIP of Pol II (rabbit polyclonal N-20; sc-899) and STAT3 (rabbit polyclonal C-20; sc-482) were from SantaCruz Biotechnology. The ChIP-grade rabbit polyclonal anti-Sp1 antibody (clone 07-645) and the monoclonal mouse anti-NF-kB p65 (RelA; clone CS2043599) were from Millipore. Mouse anti-rabbit IgG-HRP (sc-2357) and goat anti-mouse IgG-HRP (sc-2005) were from SantaCruz Biotechnology. Mouse monoclonals anti-IFNγR1 (clone 92101) and anti-IL-10Rα (clone AF-274-NA) were from R&D systems. The LEAF purified mouse IgG1k isotype control clone MG-1-45 was from Biolegend. The rabbit polyclonal anti-SMAD2/3 (clone 3102) was from Cell Science Technology.

### Primary cells and cell lines

Human monocytes were obtained from healthy donor buffy coats, upon approval by Humanitas Research Hospital Ethical Committee. Cells were isolated by two-steps gradient centrifugation using Ficoll (Biochrom) and Percoll (Amersham) followed by incubation of purified cells in RPMI 1640 (Lonza) without serum for 10 min at 37°C with 5% CO_2_. Adherent monocytes were washed twice with PBS and then cultured in RPMI medium supplemented with 10% fetal bovine serum (FBS; Lonza), 100 U/mL penicillin/streptomycin (Lonza), and 2 mM L-glutamine (Lonza). Monocyte purity was >90% as assessed by CD14/CD16 flow cytometry analysis. The human THP-1 cells (ATCC) were maintained by twice weekly passages in RPMI 1640 medium containing 10% heat-inactivated FBS, 100 U/mL penicillin-streptomycin, 25 mM L-glutamine at 37°C with 5% CO_2_. HEK-293T cells (ATCC) were grown in DMEM medium (Cambrex) supplemented with 10% heat-inactivated FBS, 100 U/mL penicillin-streptomycin, and 25 mM L-glutamine at 37°C with 5% CO_2_.

### Quantification of miRNAs and coding transcripts

Total RNA was isolated from cell cultures with TRIzol (Ambion) according to the manufacturer's instructions, and was quantified at nanodrop by its absorption at 260 nm. The A260/A230 ratio was checked to estimate nucleic acid purity. Agarose gel electrophoresis was performed using 1 μ extracted RNA to assess its integrity and evaluate the presence of contaminant genomic DNA. For quantification of miR expression, 300 ng of total RNA were reverse transcribed using TaqMan^®^ MiRNA Reverse Transcription kit (Applied Biosystems), as previously described (14). Quantitative real-time PCR (Q-PCR) was conducted using a 7900HT Real-time PCR System. Three replicates per each experimental point were performed, and differences were assessed with a 2-tailed Student *t*-test. Results were normalized on U6 levels according to the ΔΔ cycle threshold method and where indicated they are expressed as the relative change (-fold) of the stimulated group over the control group, which was used as a calibrator. The list of oligonucleotides used is reported in Table S1.

### Constructs generation

To evaluate miR activity, the 3′UTRs of target genes were amplified from genomic DNA and cloned in the biosensor psiCHECK™-2 vector (Promega). Pre-miR-125a~99b~let7e, pre-miR-125a, pre-miR-99b, and pre-let-7e were amplified from genomic DNA and subsequently cloned in the pcDNA3 expression vector, using the pCR2.1 vector (Invitrogen) as subcloning vector. To knockdown miR expression, sponge constructs (miR-125a-5p sponge and let7e-5p sponge) containing multiple sequential repeats of miR imperfect complementary seed site regions were cloned into a psiCHECK™-2 vector (Promega). The fragment containing the SV40 promoter together with the *renilla* luciferase gene fused to the miR sponge fragment was then subcloned into the pRRLSIN.CPPT.PGK.GFP.WPRE vector (plasmid #12252; Addgene) in an antisense orientation with respect to the GFP cassette. The expression of the luciferase reporter gene was checked to assess the efficacy of miR inhibition. A lentiviral construct encoding for a hairpin yielding a 22-mer RNA designed to lack homology to any human gene was used as control.

### Luciferase reporter assay

HEK-293T cells were plated in 24-well plates in DMEM supplemented with 10% FBS and 1% of L-glutamine at 16 × 10^4^/well and after 24 h were transfected with 100 ng psiCHECK™-2-3′-UTR reporter construct and 10 μM mirVana miR mimics (Life Technologies) using Lipofectamine 2000 (Invitrogen), according to the manufacturer's protocol. After 48 h from transfection, cells were lysed and *firefly* and *renilla* luciferase activities were determined using the Dual-Glo Luciferase Assay System (Promega), according to manufacturer's instructions. The enzymatic activities of the two luciferases were quantified using a MultiDetection Microplate Reader Synergy 2 luminometer (BioTek). The values of *renilla* luciferase activity were normalized by *firefly* luciferase activity, which served as internal control, and expressed as fold changes relative to the value of the negative control.

### Chromatin immunoprecipitation (ChIP) assay

ChIP experiments were performed as previously described (14). Briefly, sheared chromatin from 5 × 10^6^ monocytes was immunoprecipitated ON at 4°C with the relative antibody and 1% of starting chromatin was used not immunoprecipitated and used as input. Q-PCR was performed in triplicates using primers reported in Table S1. Signals obtained from the ChIP samples were normalized on signals obtained from corresponding input samples, according to the formula: 100 × 2^(input Ct−sample Ct)^. Results were expressed as fold enrichment relative to untreated cells.

### Flow cytometry and surface staining

Cells were washed twice with PBS containing 1% BSA and unspecific binding was blocked using Fc-block (BD Biosciences). Washed cells were resuspended in a 1:200 dilution of APC-conjugated anti-human TLR4 antibody (clone HTA125 from eBioscience), anti-human CD14 antibody (clone 61D361 from eBioscience) or the mouse APC-conjugated IgG2a isotype control (eBioscience) and incubated for 1 h at 4°C. Stained cells were washed twice with PBS containing 1% BSA and then analyzed by flow cytometry (FACS Canto, BD Biosciences).

### LPS desensitization

Monocytes were cultured in 24-well plates in 500 μl RPMI supplemented with 10% FBS and 1% L-glutamine, pretreated or not ON with 10 ng/ml IFNγ, primed or not with 0.1 ng/ml LPS for 18 h, and then challenged with 10 ng/ml LPS. THP-1 cells, transduced with miRT-125a-5p sponge or control vectors, were cultured in 24-well plates in 500 μl RPMI supplemented with 10% FBS and 1% L-glutamine, pretreated or not ON with 10 ng/ml IFNγ, primed or not with 50 ng/ml TGFβ or 10 ng/ml IL-10 for 2 h, and then challenged with 100 ng/ml LPS. In both experimental settings, cell lysates and supernatants were collected at 48 h.

### Immunoprecipitation of Ago2-bound RNAs (RIP)

RIP experiments were performed as previously described (14, 20). Briefly, immunoprecipitations were carried out ON at 4°C using protein G sepharose magnetic beads (GE Healthcare) conjugated with anti-Ago2 (EIF2C2 monoclonal antibody clone 2E12-1C9; Abnova) or an isotype IgG1k control antibody (Abnova). Sequences of 3'UTR mRNA-specific primers used in Q-PCR are listed in Table S1. After immunoprecipitation an aliquot of supernatants corresponding to 0.5 × 10^6^ cell equivalent (indicated as “leftover”) was removed and used as control for the specificity of the assay. The miR/mRNA enrichment to the RNA-induced silencing complex (RISC) complex was calculated according to the formula: 2^−(CtAgo−CtIgG)^. Results were expressed as fold enrichment relative to Ago2-IP CT samples.

### Western blot analysis

Protein lysates and Western blots were performed as previously described (19). Transfected THP-1 cells (5 × 10^6^) were harvested, incubated at 4°C for 5 min with 1 mM diisopropyl fluorophosphate, and lysed in 40 mM Tris, 1% SDS, 7.5% glycerol. 100 μg of the total cell lysate was boiled for 10 min at 95°C, resolved on 10% SDS-PAGE, and transferred to nitrocellulose (Hybond; GE Healthcare). Blots were incubated with anti-IRAK1 (clone D51G7 from Cell Science Technology) and anti-β actin (clone C4; sc-47778 from SantaCruz Biotechnology) antibodies and then probed with appropriate HRP-conjugated secondary antibodies (donkey anti-rabbit IgG LNA934V/AG and sheep anti-mouse IgG LNXA931/AE from Amersham, respectively).

### ELISA assay

Cytokine levels (TNFα, CCL3, CXCL8, IL-6, IL-12p40, CCL7, CCL2, CXCL10) present in the cell culture supernatant of transduced THP-1 cells stimulated for the indicated times with LPS or IFNγ were measured using the human Duoset ELISA kit (R&D Systems), according to the manufacturer's instructions. Samples were diluted so that the optical density fell within the optimal portion of a log standard curve.

### Bioinformatics analysis

Predicted target genes of each miR were defined using the microrna.org database (21). The enrichment analysis of GO terms was performed through the analysis tool available from the Panther Classification System (22). Statistical overrepresentation test (Bonferroni test) was performed using default setting. The analysis of biological functions and associated networks was determined using the Ingenuity Pathway Analysis software (IPA; Ingenuity Systems) by applying the “expression in immune cells” filter and the built-in Fisher exact test. The relationship of the miR-125a~99b~let-7e cluster with the 124 genes included in the Inflammatory response network shown in Figure 6 was graphically visualized using IPA. The probability score of each the miR-125a~99b~let-7e cluster to be involved in this network was calculated according to the formula: miRx = −1/log2[(Txϵ N)/124], where T = number of predicted target genes of miRx and N = number of genes included in the Inflammatory response network. The statistical value of the involvement in the TLR signaling pathway, as identified by IPA, was defined by fitting the target distribution to Gaussian functions with mean 25.74 and SD 11.40. Evolutionary conserved regions and transcription factor binding sites were identified using the Mulan software (http://mulan.dcode.org) (23) and visualized in Figure 2 using the Jalview 2.8 software (www.jalview.com) (24).

### Statistical analysis

Statistical evaluation was determined using the Student *t*-test or the one-way ANOVA and *p*-values are reported in figures (^*^ <0.05; ^**^ <0.01; ^***^ <0.001).

## Results

### The miR-125a~99b~let-7e cluster is induced by IL-10 and TGFβ

**Figure 1:**
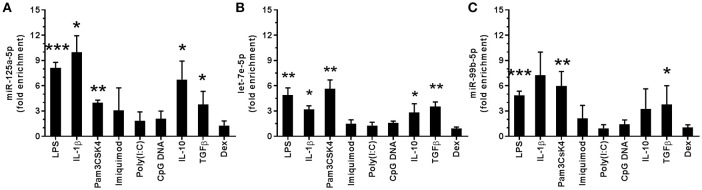
Regulation of the miR-125a~99b~let-7e cluster expression. Expression of miR-125a-5p, let-7e-5p, and miR-99b-5p in monocytes cultured for 24 h with 100 ng/mL LPS, 2 μg/mL pam3CSK4, 3 μg/mL imiquimod, 50 μg/mL poly(I:C), 1 μM CpG DNA, 25 ng/mL IL-1β, 50 ng/mL IL-10, 50 ng/mL TGFβ, 20 ng/mL Dex **(A–C)**. MiR-125a-5p **(A)**, let-7e-5p **(B)** and miR-99b-5p **(C)** were measured by Q-PCR in triplicate samples. Results expressed as fold change over control (mean ± SEM; *n* = 4). (* < 0.05; ** < 0.01; *** < 0.001).

**Figure 2:**
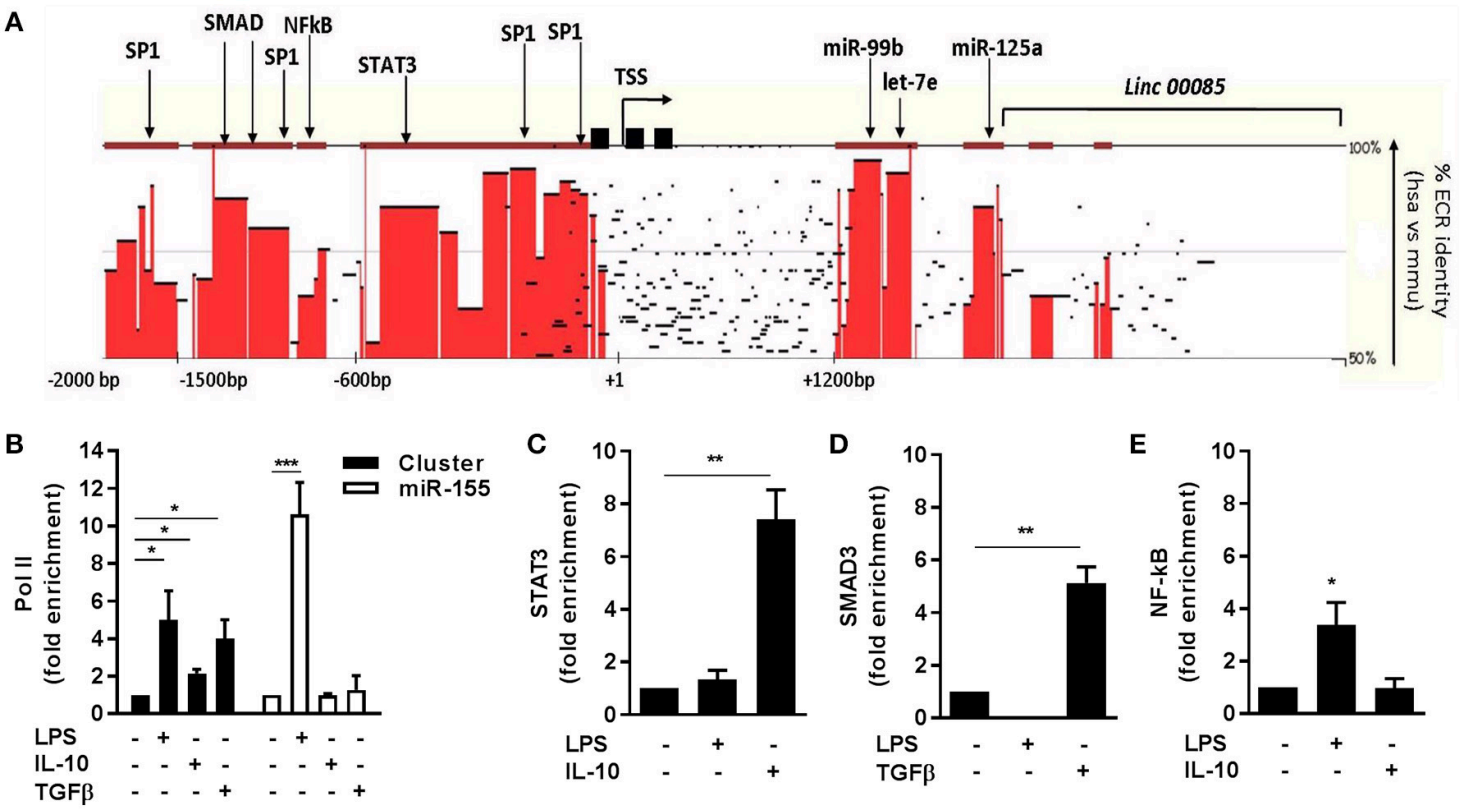
Transcriptional regulation of miR-125a~99b~let-7e cluster. **(A)** Sequence homology between human (chr.19: 52191745-52196592) and murine (chr. 17:17827604-17832451) miR-125a~99b~let-7e *loci* was analyzed by pairwise sequence local alignment using the TBA alignment program and visualized as a conservation plot generated by the Mulan software. Red blocks identify evolutionary conserved regions (ECR) with >50% identity between the two species over 100 bp segments. The transcription start site (TSS), TATA boxes (black squares), and the putative binding sites for transcription factors are indicated. **(B–E)** ChIP assays were carried out on human monocytes stimulated or not for 4 h with 25 ng/ml IL-10, 100 ng/ml LPS or 50 ng/ml TGFβ using anti-Pol II **(B)** Abs or for 12 h with anti-STAT3 **(C)**, anti-SMAD3 **(D)**, or anti-NF-kB **(E)** antibodies. Q-PCR was carried out using specific primers for the miR-125a~99b~let-7e promoter (black columns in panels **B–E**) or the miR-155 promoter (white columns in panel **B**). Results are expressed as fold change over control (mean ± SEM; *n* = 3). (* < 0.05; ** < 0.01; *** < 0.001).

To identify miR potentially involved in the response to stimuli of bacterial origin, we have previously analyzed the miR expression profile of monocytes stimulated with 100 ng/ml of the TLR4 agonist LPS (8). Under these conditions, we reported that the panel of LPS-induced miRs included miR-125a-5p, let-7e-5p, and miR-99b-5p (Figures 1A–C). These three miR represent the mature products of the miR-125a~99b~let-7e cluster, encoded by a conserved region hosted in the first intron of the *linc 00085* gene in human chromosome 19 (Figure 2A). This miR cluster was induced to similar extent to LPS also by the TLR2 agonist pam3CSK4 and the proinflammatory cytokine IL-1β, while agonists at intracellular TLRs, such as the TLR3 agonist poly (I:C), the TLR7 agonist imiquimod, and a CpG oligodeoxynucleotide (CpG DNA oligo) agonist at TLR9, were inactive (Figures 1A–C). Interestingly, when other anti-inflammatory mediators were tested, TGFβ and IL-10 were also able to increase the expression of miR-125a~99b~let-7e cluster, while glucocorticoids (Dex) were inactive (Figures 1A–C).

The transcriptional regulation of the miR-125a~99b~let-7e cluster was further investigated by evaluating by ChIP the engagement of cis regulatory elements on predicted binding sites included in its putative proximal promoter region, located by bioinformatics analysis within 2 kb upstream its transcription start site (TSS) (Figure 2A). The increased expression of the miR cluster observed upon LPS challenge was accompanied by a comparable recruitment of Pol II at the core promoter region of the miR-125a~99b~let-7e cluster and also at the promoter of miR-155, a well-known LPS-responsive miR used as a positive control (25) (Figure 2B). Conversely, after exposure to IL-10 or TGFβ, monocytes showed Pol II recruitment on the promoter region of the miR-125a~99b~let-7e cluster but not on the miR-155 promoter, here used as a negative control being negatively regulated by IL-10 (17) (Figure 2B). Consistent with the positive effect of IL-10 and TGFβ on the expression of the miR-125a~99b~let-7e cluster, binding of the IL-10-dependent transcription factor STAT3 and TGFβ-dependent transcription factor SMAD3 to highly conserved binding sites was also evident after short-term exposure to IL-10 and TGFβ, respectively (Figures 2C,D). Interestingly, LPS but not IL-10 also induced engagement of the transcription factor NF-kB, suggesting its involvement on miR induction after LPS (Figure 2E).

**Figure 3:**
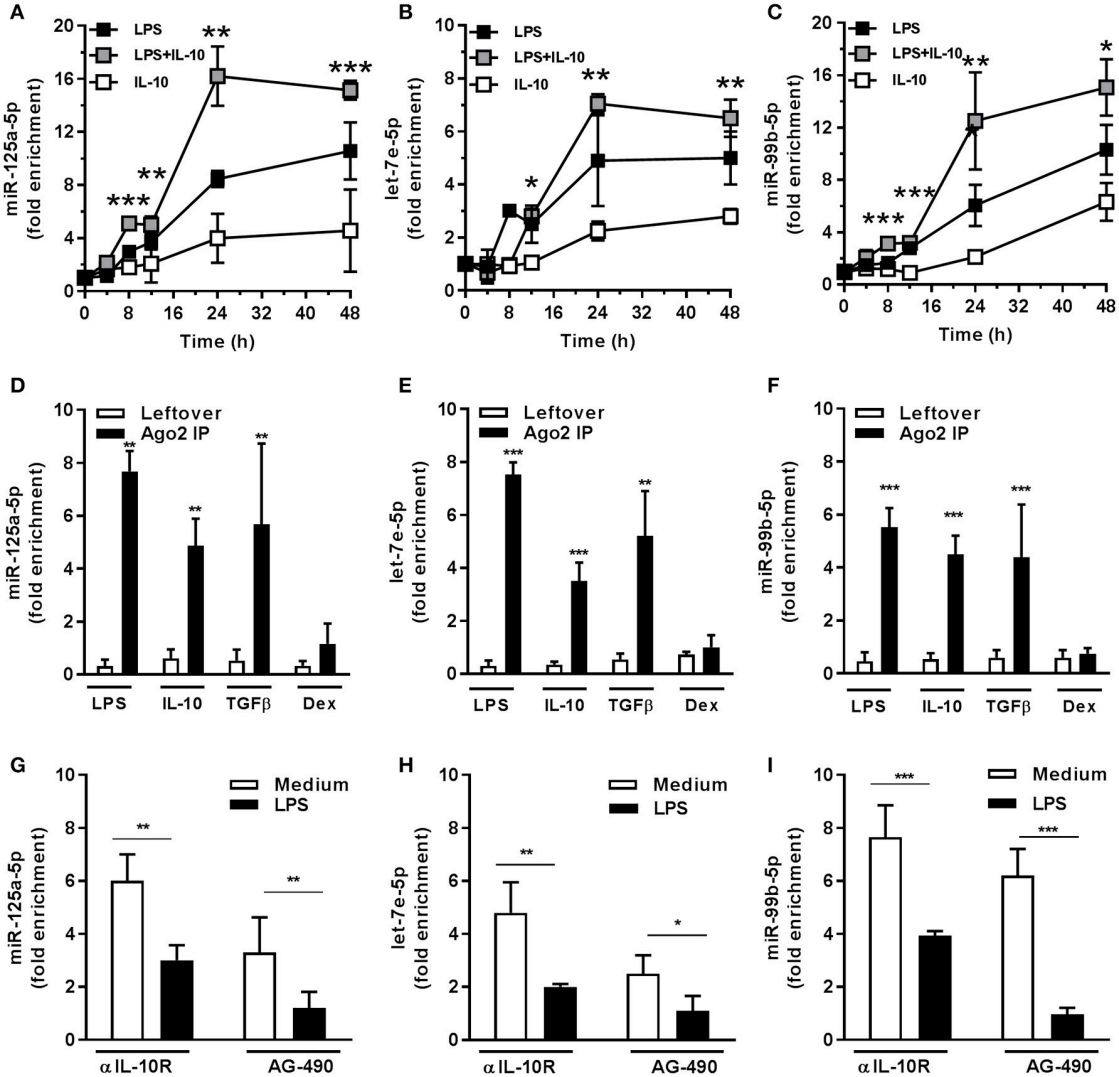
miR-125a~99b~let-7e cluster is induced through an IL-10-mediated autoregulatory loop. **(A–C)** Monocytes were treated for the indicated time points with 100 ng/mL LPS (black), 25 ng/mL IL-10 (white), or both stimuli (gray). Results expressed as fold change over unstimulated samples (mean ± SEM; *n* = 4). **(D–F)** Monocytes were cultured with 100 ng/mL LPS, 50 ng/mL IL-10, 50 ng/mL TGFβ and 20 ng/mL Dex for 24 h and cell extracts were subjected to RIP assay using anti-Ago2 or IgG control Abs. MiR-125a-5p **(A,D)**, let-7e-5p **(B,E)**, and miR-99b-5p **(C,F)** were measured by Q-PCR in triplicate samples. Results expressed as fold change over unstimulated samples (mean ± SEM; *n* = 6). **(G–I)** Monocytes were stimulated for 12 h with 100 ng/mL LPS after pre-treatment for 30 min with the JAK/STAT inhibitor AG-490 (5 μM) or its vehicle (black and white columns, respectively) or in the presence of anti-IL-10R (10 μg/mL) or isotype control MoAb (black and white columns, respectively). MiR-125a-5p **(G)**, let-7e-5p **(H)**, and miR-99b-5p **(I)** were measured by Q-PCR in triplicate samples. Results expressed as fold change over control (mean ± SEM; *n* = 6). (* < 0.05; ** < 0.01; *** < 0.001).

**Figure 4:**
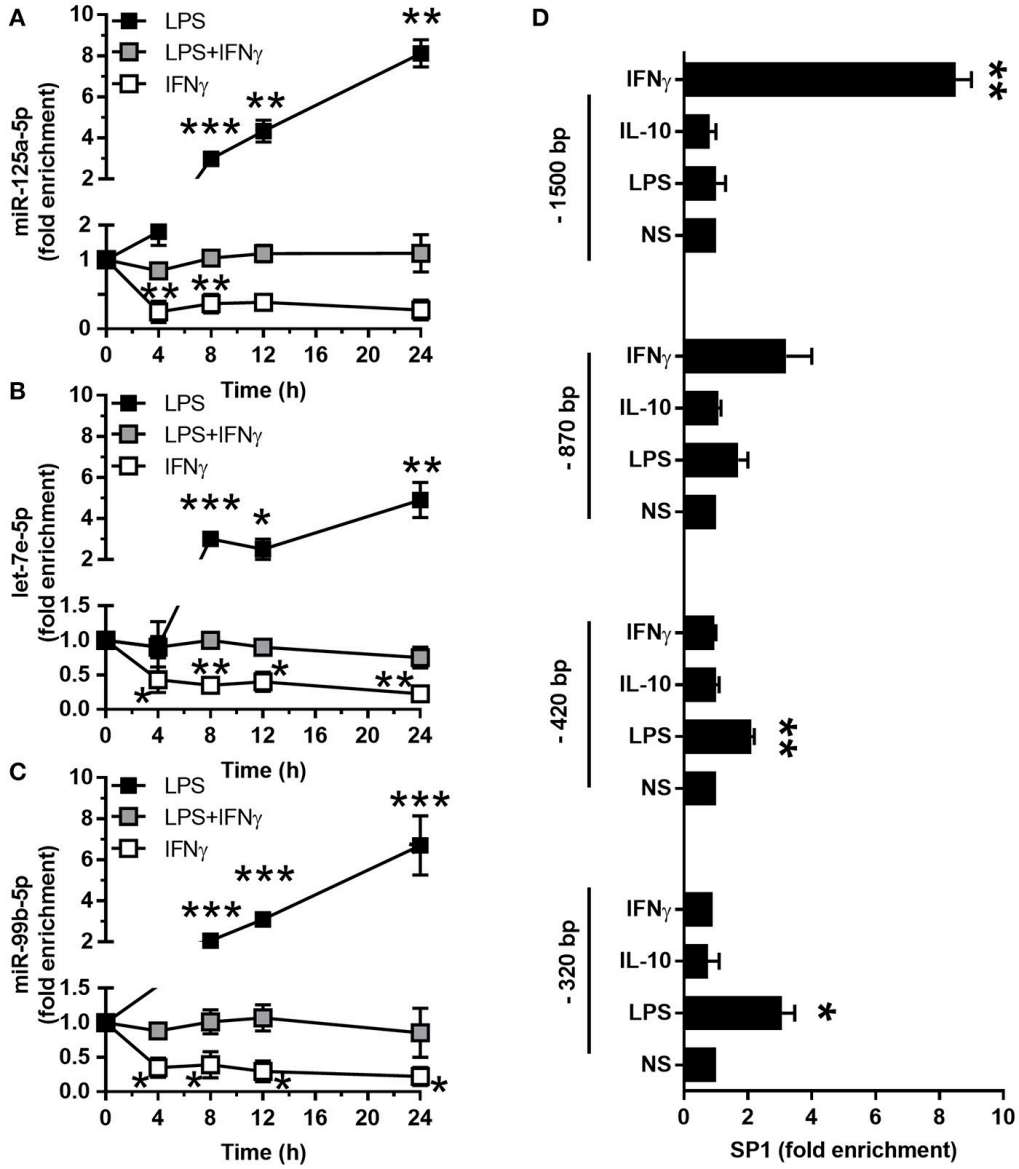
Interferon antagonizes the induction of miR-125a~99b~let-7e cluster**. (A–C)** Expression of miR-125a-5p, let-7e-5p and miR-99b-5p in monocytes cultured for 24 h with 100 ng/mL LPS (black), 20 ng/mL IFNγ (white) or both stimuli (gray) for the indicated times. MiR-125a-5p **(A)**, let-7e-5p **(B)**, and miR-99b-5p **(C)** were measured by Q-PCR in triplicate samples. Results expressed as fold change over control (mean ± SEM; *n* = 4). **(D)** ChIP assays were carried out on monocytes stimulated or not for 24 h with 25 ng/ml IL-10, 100 ng/ml LPS, or 10 ng/ml IFNγ using a monoclonal antibody recognizing SP1. Q-PCR was carried out using primers specific for the predicted SP1 binding sites in the miR-125a~99b~let-7e promoter, located at the distances from the TSS indicated in Figure 2A. Results are expressed as fold change over control (mean ± SEM; *n* = 4). (* < 0.05; ** < 0.01; *** < 0.001).

The LPS-dependent induction of the miR-125a~99b~let-7e cluster occurred with a delayed kinetic as compared to other miRs directly induced by LPS, such as miR-155 (8, 25), suggesting the involvement of second mediators (Figures 3A–C). Indeed IL-10, which is late induced in monocytes after LPS exposure (2, 26, 27), was able to upregulate miR-125a-5p, miR-99b-5p, and let-7e-5p expression (Figures 1A–C, 3A–C) and potentiated their induction by LPS (Figures 3A–C). Of note, LPS, IL-10, and TGFβ, but not Dex, also induced an enrichment of mature miRs into the RNA-induced silencing complex (RISC) complex (Figures 3D–F), indicating that these miRs are functionally active in monocytes. Furthermore, when the activity of the endogenous IL-10 late induced after TLR4 engagement was blocked, either by an anti-IL-10R blocking monoclonal antibody or the JAK/STAT signaling pathway inhibitor AG-490 compound (28), a significant reduction of miR-125a-5p, miR-99b-5p, and let-7e-5p induction by LPS was observed (Figures 3G–I). IFNγ is a key mediator of chronic inflammation and is known to show positive synergistic effects with LPS and negative synergism with IL-10 (4). Consistently, treatment with IFNγ reduced miR-125a~99b~let-7e expression at early time-points and abolished its induction by the IL-10-dependent loop observed at late time-points after LPS stimulation (Figures 4A–C). Of note, upon stimulation IFNγ efficiently recruited the SP1 trancription factor at the most distal binding site on the cluster promoter (Figure 4D). Taken together, these data identify the miR-125a~99b~let-7e cluster as an IL-10 and TGFβ responsive gene, negatively modulated by IFNγ, and suggest its involvement in the complex feedback regulatory mechanism controlling the inflammatory response after TLR engagement.

### MiR-125a-5p and let-7e-5p directly target the TLR signaling pathway at multiple levels

**Figure 5:**
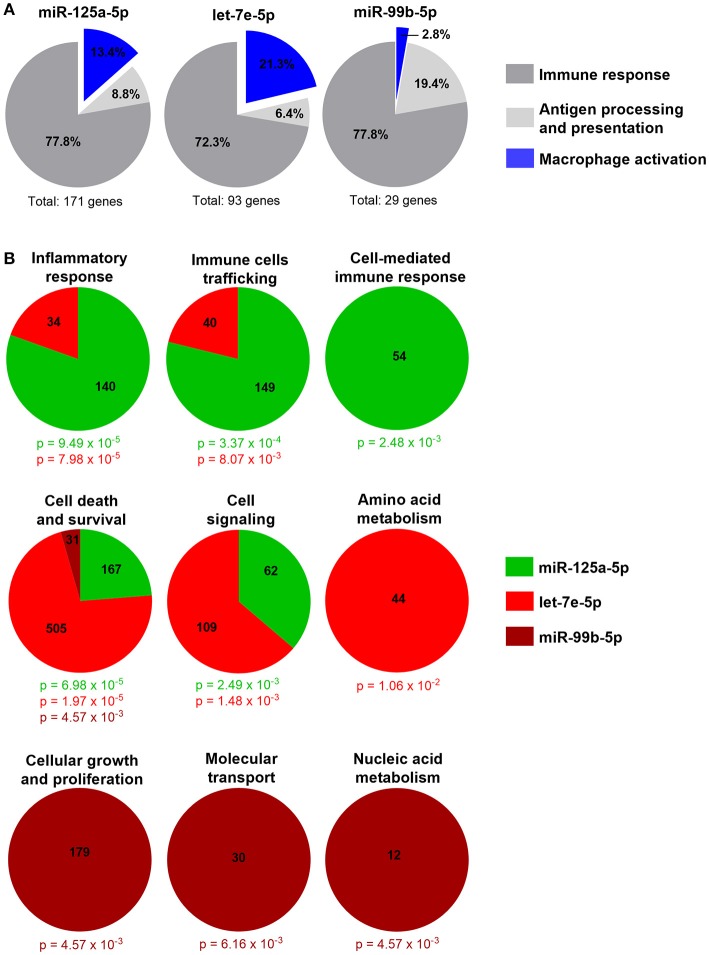
Inflammatory genes are enriched in miR-125a~99b~let-7e cluster predicted target genes**. (A)** Absolute number and percentage of miR-125a-5p, let-7e-5p, and miR-99b-5p predicted target genes included in the *Immune system process* genonthology category (GO term 0002376) and in the relative subcategories *Immune response* (dark gray), *Antigen processing and presentation* (light gray), and *Macrophage activation* (blue). **(B)** Molecular and cellular functions significantly enriched in predicted target genes of miR-125a-5p (green), let-7e-5p (red), and miR-99b-5p (brown) as identified by the IPA analysis. The number of molecules involved and the *p* value calculated with the right-tailed Fisher's exact test are reported in the color code corresponding to each miR.

**Figure 6:**
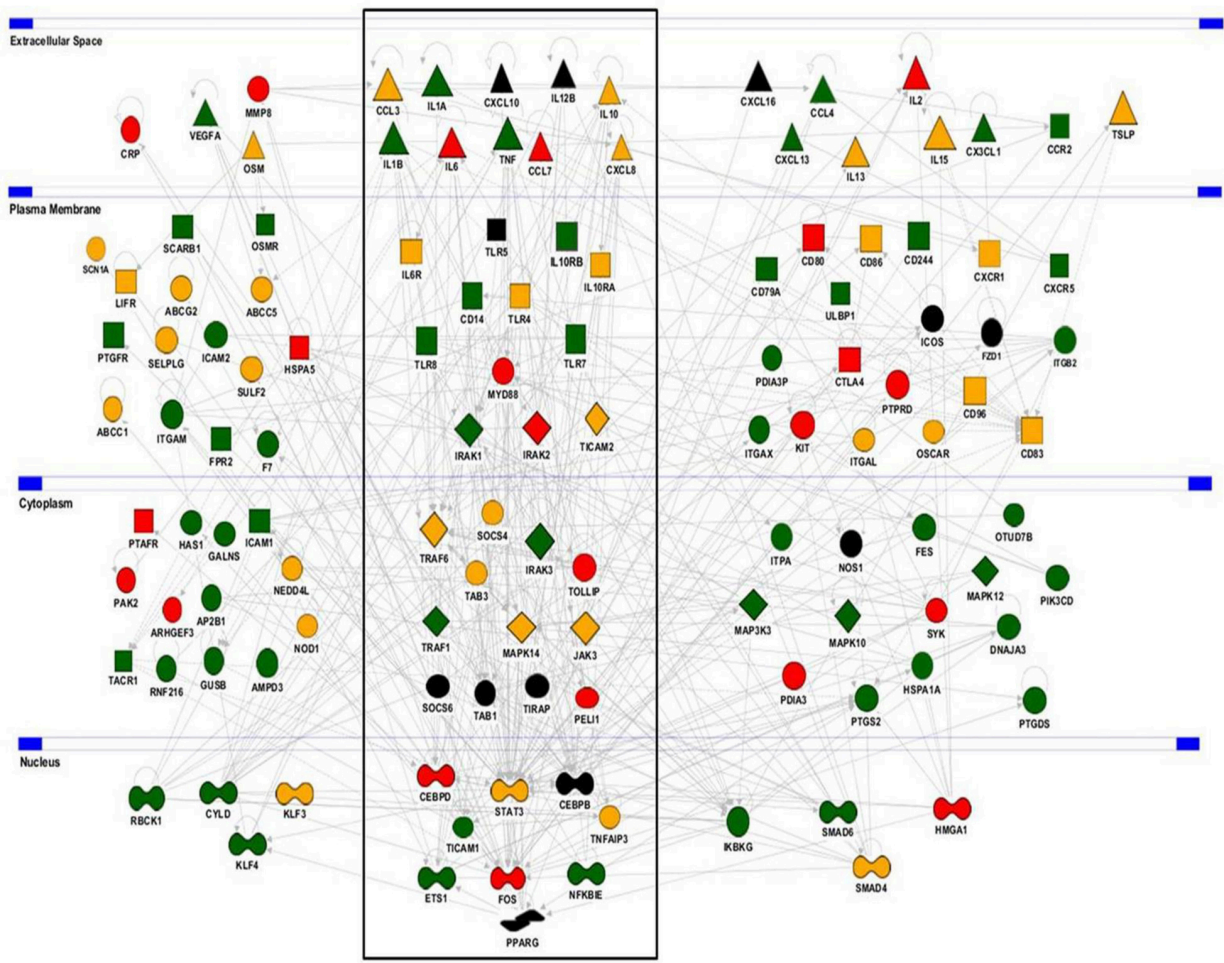
Predicted target genes of miR-125a-5p and let-7e-5p in the *Inflammatory response* molecular network. The Inflammatory response molecular network was extracted using the IPA analysis knowledge database and used to display functional relationships with predicted target genes of miR-125a-5p (green), let-7e-5p (red), or both (yellow). Genes not predicted as targets are in black. The *Toll-Like receptor pathway* (boxed) shows a significant enrichment of genes predicted as direct targets of the miR-125a~99b~let-7e cluster (*p* = 1.42 × 10^−5^).

**Figure 7:**
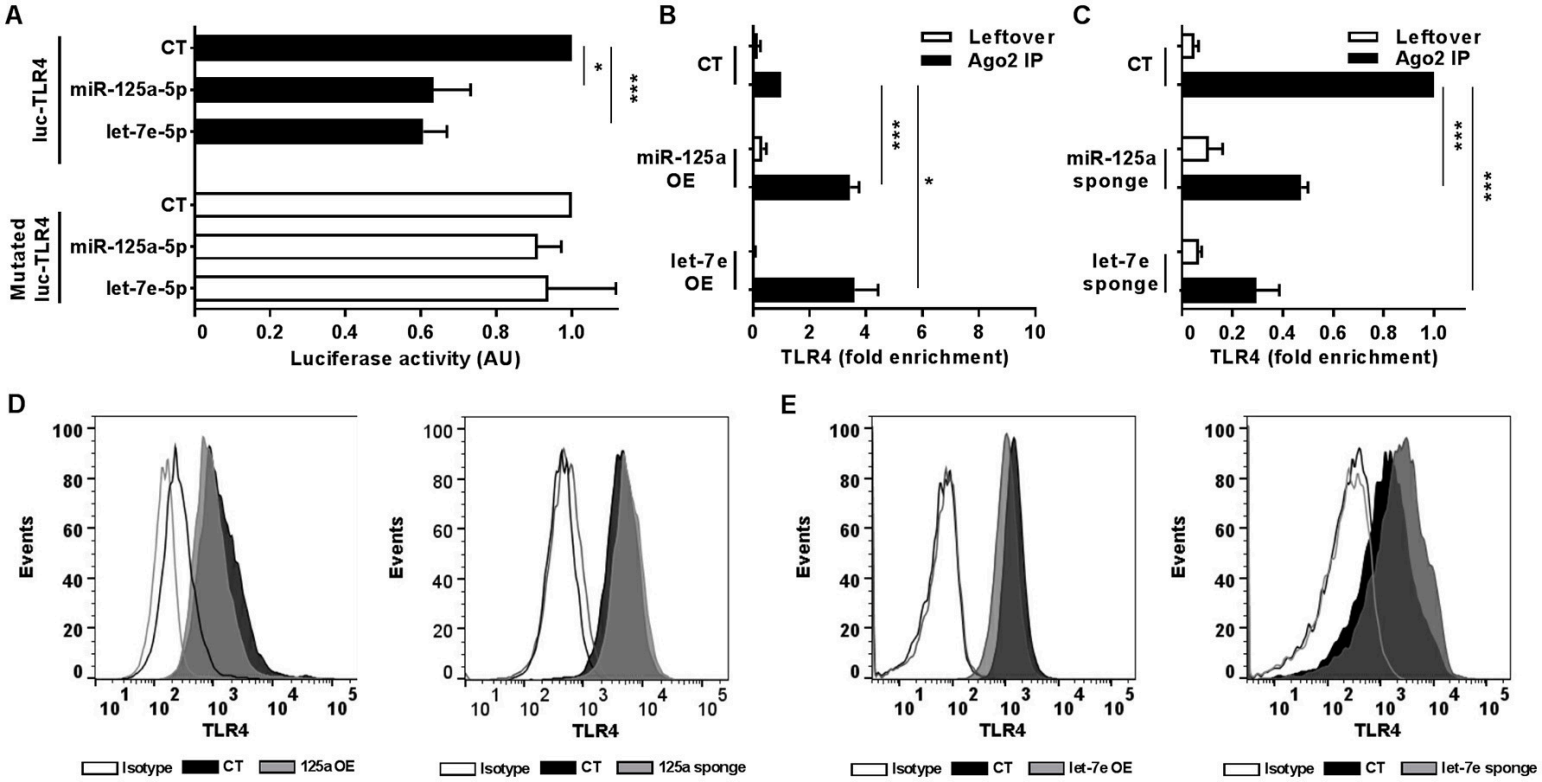
MiR-125a-5p and let-7e-5p directly target TLR4. **(A)** Wild-type or MRE-mutated luciferase constructs based on the 3′UTR of TLR4 were cotransfected in HEK-293T cells with mimics of miR-125a-5p, let-7e-5p, miR-99b-5p, or a negative control (CT). Results are expressed as mean (% variation ± SEM; *n* = 6) of the ratio between *renilla* luciferase and *firefly* control luciferase activities. **(B,C)** Cell extracts from THP-1 cells transduced with miR-125a OE, let-7e OE, or the control vector CT **(B)** or with miR-125a-5p sponge, let-7e-5p sponge, or the control vector CT **(C)** were stimulated for 6 h with LPS and then subjected to RIP assay using anti-Ago2 or IgG control Abs. TLR4 transcript levels were assayed in triplicate by Q-PCR (mean ± SEM; *n* = 6) and expressed as normalized fold enrichment in Ago2 IP (black columns) and leftover (white columns). **(D–E)** Surface molecule protein levels were measured by flow cytometry in THP-1 cells transduced with over-expressing or sponge vectors (gray bars on the left and right panels, respectively) for miR-125a **(D)** or let-7e **(E)** or their corresponding control vectors (black bars). Isotype control staining are shown in dotted histograms. Results from one representative experiment of 3 performed are shown. (* < 0.05; *** < 0.001).

**Figure 8:**
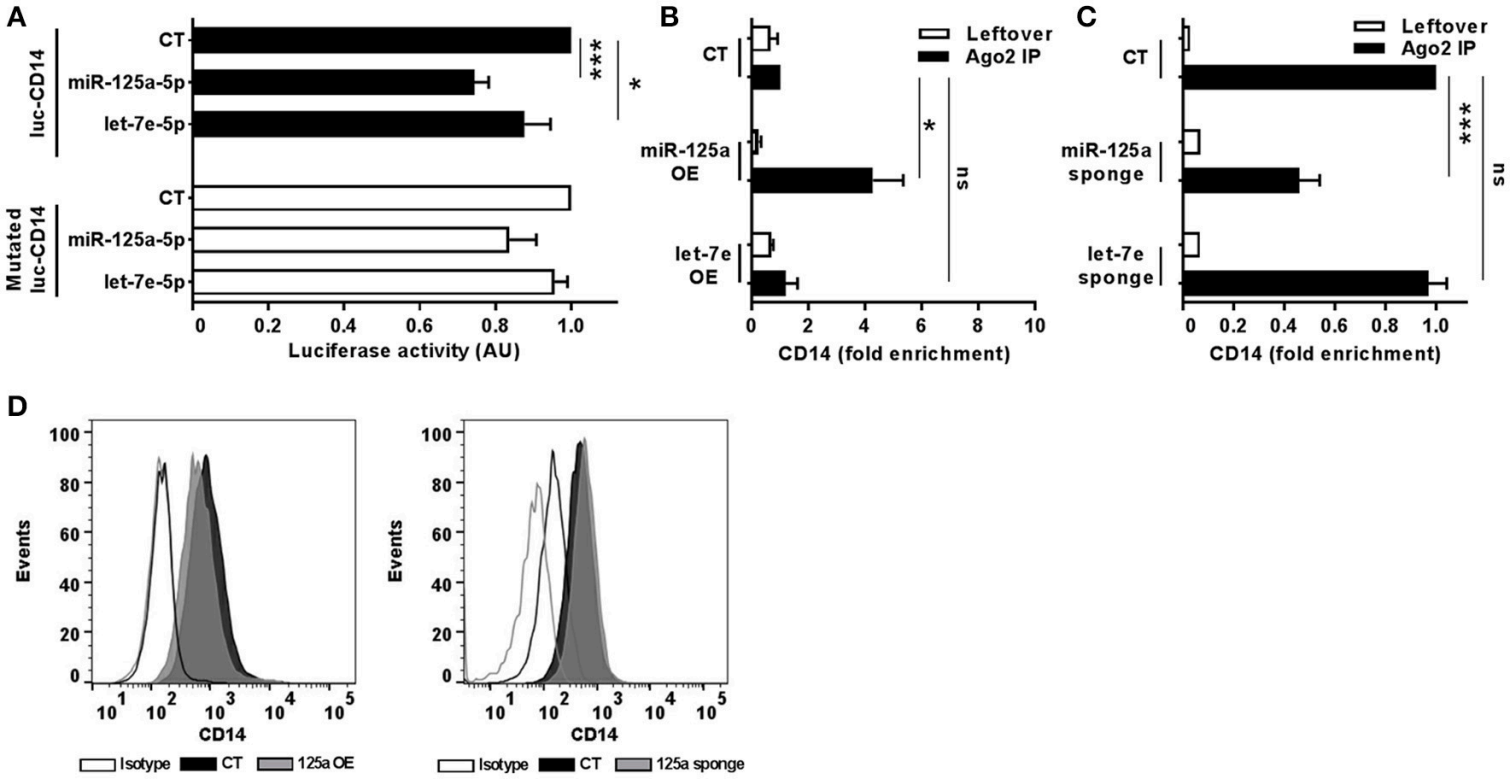
MiR-125a-5p directly targets CD14. **(A)** Wild-type or MRE-mutated luciferase constructs based on the 3'UTR of CD14 were cotransfected in HEK-293T cells with mimics of miR-125a-5p, let-7e-5p, miR-99b-5p, or a negative control (CT). Results are expressed as mean (% variation ± SEM; *n* = 6) of the ratio between *renilla* luciferase and *firefly* control luciferase activities. **(B,C)** Cell extracts from THP-1 cells transduced with miR-125a OE, let-7e OE, or the control vector CT **(B)** or with miR-125a-5p sponge, let-7e-5p sponge, or the control vector CT **(C)** were stimulated for 6 h with LPS and then subjected to RIP assay using anti-Ago2 or IgG control Abs. CD14 transcript levels were assayed in triplicate by Q-PCR (mean ± SEM; *n* = 6) and expressed as normalized fold enrichment in Ago2 IP (black columns) and leftover (white columns). **(D)** Surface molecule protein levels were measured by flow cytometry in THP-1 cells transduced with over-expressing or sponge vectors (gray bars on the left and right panels, respectively) for miR-125a or the corresponding control vectors (black bars). Isotype control staining are shown in dotted histograms. Results from one representative experiment of 3 performed are shown. (* < 0.05; *** < 0.001).

**Figure 9:**
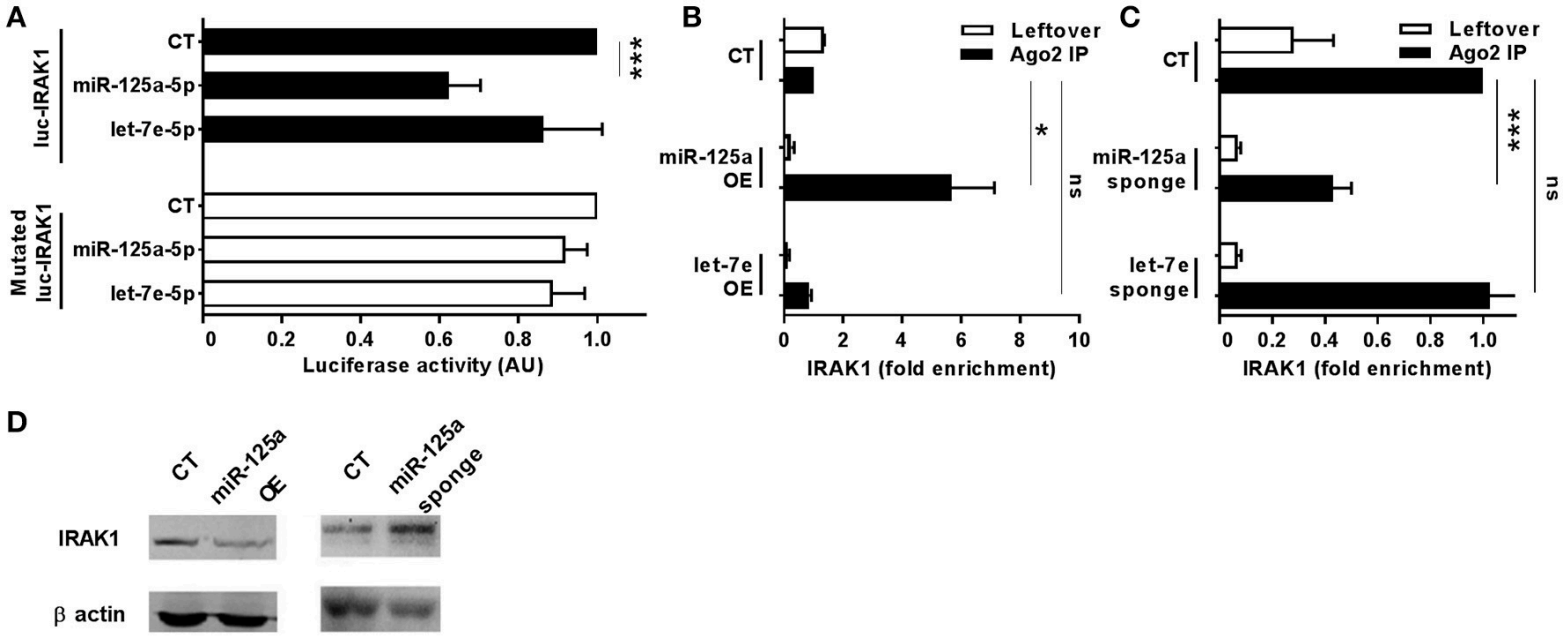
MiR-125a-5p directly targets IRAK1. **(A)** Wild-type or MRE-mutated luciferase constructs based on the 3′UTR of IRAK1 were cotransfected in HEK-293T cells with mimics of miR-125a-5p, let-7e-5p, miR-99b-5p, or a negative control (CT). Results are expressed as mean (% variation ± SEM; *n* = 6) of the ratio between *renilla* luciferase and *firefly* control luciferase activities. **(B,C)** Cell extracts from THP1 cells transduced with miR-125a OE, let-7e OE, or the control vector CT **(B)** or with miR-125a-5p sponge, let-7e-5p sponge, or the control vector CT **(C)** were stimulated for 6 h with LPS and then subjected to RIP assay using anti-Ago2 or IgG control Abs. IRAK1 transcript levels were assayed in triplicate by Q-PCR (mean ± SEM; *n* = 5) and expressed as normalized fold enrichment in Ago2 IP (black columns) and leftover (white columns). **(D)** IRAK1 protein levels were evaluated by Western blot in THP-1 cells transduced with over-expressing or sponge vectors (left and right panels, respectively) for miR-125a. Normalization was performed on β actin levels (lower panels) evaluated on the same blot. Results from one representative experiment of 3 performed are shown. (* < 0.05; *** < 0.001).

To uncover the biological functions of miRs encoded by this cluster and to gain insights into their functional role in the context of LPS-mediated inflammation, we examined the gene onthology categories associated to the predicted target genes of the three miRs. A significant enrichment of genes involved in the *Immune system* process (GO term 0002376) was observed for miR-125-5p and let-7e-5p (miR-125a-5p: 171 genes, *p* = 3.52 × 10^−3^; let-7e-5p: 93 genes, *p* = 2.11 × 10^−2^) but not miR-99b-5p (29 genes, *p* = 0.787). Of note, the list of predicted targets associated to this specific genonthology cathegory showed in particular a highly significant enrichment of the functional annotation *Macrophage activation* (GO term 0042116) for miR-125-5p and let-7e-5p, but not for miR-99b-5p (Figure 5A). Consistent with this, the intersection between the predicted targets of each miR of the cluster and biological pathways as defined by IPA indicated a significant enrichment of genes associated to biological functions related to inflammation (*Inflammatory response, Immune cell trafficking, Cell-mediated immune response*) for miR-125a-5p and, to a less extent, let-7e-5p, but not miR-99b-5p, which was not related to any of these functions (Figure 5B). Finally, the IPA analysis of the predicted target genes of miR-125a-5p and let-7e-5p generated an “inflammatory network” centered on the TLR pathway, including receptors (TLR4, CD14), signaling molecules (IRAK1), and inflammatory mediators (TNFα, IL-6, CCL3, CCL7, CXCL8), and linked to a set of nodes with higher connectivity as compared to the rest of the network (Figure 6). Consistent with targeting predictions, over-expression of miR-125a-5p or let-7e-5p significantly decreased the luciferase activity of a reporter construct containing the TLR4 3′UTR, and a 5-bp deletion in the miR-125a-5p or let-7e-5p MRE fully restored luciferase levels, indicating the specificity of these miRs for their predicted target sites in the TLR4 transcript (Figure 7A). When the human monocytic cell line THP-1, a well-established model for *in vitro* studies of the TLR signaling pathway (29), was transduced with lentiviral vectors over-expressing miR-125a or let-7e (miR-125a OE and let-7e OE, respectively), a significant enrichment of the TRL4 transcript in the RISC was observed, as compared to cells transduced with the control vector (CT) (Figure 7B). In a complementary approach, cells transduced with lentiviral vectors expressing artificial mRNA targets to inhibit miR-125a-5p or let-7e-5p (miR-125a-5p sponge and let7e-5p sponge, respectively) showed a significant decrease in the TLR4 transcript enrichment in the RISC after LPS stimulation as compared to cells transduced with the control vector (CT) (Figure 7C). Finally, when compared to control vector-transduced cells, TLR4 protein levels were significantly decreased in cells transduced with miR-125a OE and let7e OE and significantly increased in cells transduced with miR-125a-5p sponge and let7e-5p sponge (Figures 7D,E). Taken together, these data validated the predicted direct targeting of TLR4 by both miR-125a-5p and let7e-5p. Using a similar approach, the direct targeting of the second LPS receptor CD14 and the TLR signaling molecule IRAK1 were also demonstrated for miR-125a-5p but not let7e-5p, consistent with targeting predictions (Figures 8A–D, 9A–D, respectively).

**Figure 10:**
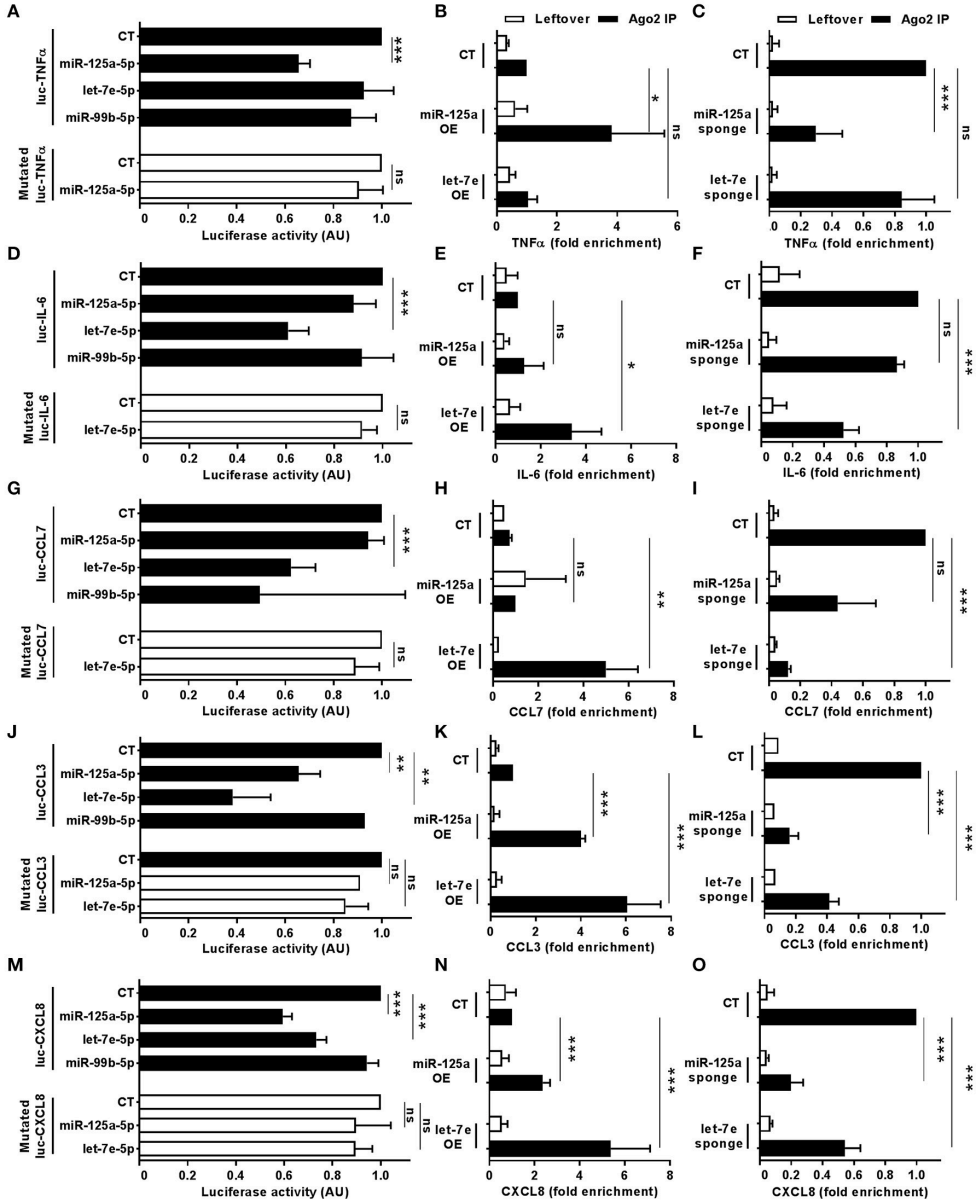
Direct targeting of pro-inflammatory cytokines by miR-125a-5p and let-7e-5p. Wild-type of MRE-mutated luciferase constructs based on the 3'UTR of TNFα **(A)**, IL-6 **(D)**, CCL7 **(G)**, CCL3 **(J)**, and CXCL8 **(M)** were cotransfected in HEK-293T cells with miR-125a-5p, let-7e-5p, miR-99b-5p mimics or with a negative control mimic. Results are expressed as mean (% variation ± SEM; *n* = 5) of the ratio between *renilla* luciferase and *firefly* control luciferase activities. Cell extracts from THP1 cells transduced with miR-125a OE, let-7e OE, and the control vector (CT; panels **B,E,H,K,N**) or with miR-125a-5p sponge, let-7e-5p sponge, and the control vector (CT; panels **C,F,I,L,O**) were stimulated for 6 h with LPS and then subjected to RIP assay using anti-Ago2 or IgG control Abs and levels of TNFα **(B,C**), IL-6 **(E,F)**, CCL7 **(H,I)**, CCL3 **(K,L**), and CXCL8 **(N,O**). Transcript levels were assayed in triplicate by Q-PCR (mean ± SEM; *n* = 4) and expressed as normalized fold enrichment in Ago2 IP (black columns) and leftover (white columns). (* < 0.05: ** < 0.01; *** < 0.001).

Bioinformatics predictions of miR-125a-5p and let-7e-5p targets also included a relevant number of LPS-dependent pro-inflammatory cytokines (Figure 6; green and red indicate miR-125a-5p and let-7e-5p target genes, respectively, targets of both miRs are in yellow). Luciferase assays validated the TNFα transcript as a direct target of miR-125a-5p, and mutations at the corresponding MRE abolished miR125a-5p-mediated suppression, demonstrating sequence specificity of the targeting (Figure 10A). Consistent with this, RIP analysis revealed a significant enrichment of the TNFα transcript in miR-125a OE transduced THP-1 cells (Figure 10B) and a parallel decrease of the TNFα transcript in the RISC when miR-125a-5p was inhibited (Figure 10C). Similar approaches confirmed IL-6 and CCL7 targeting by let-7e-5p (Figures 10D–I, respectively), while CCL3 and CXCL8 were validated as targets of both miR-125a-5p and let-7e-5p (Figures 10J–O, respectively). Expression of miR-125a-5p and let-7e-5p caused a significant reduction of most but not all their direct target mRNA, indicating that depending on the specific transcript either mRNA destabilization or transduction inhibition could be involved in their inhibitory effect (Figure S1).

### The miR-125a~99b~let-7e cluster inhibits LPS-dependent production of inflammatory cytokines

**Figure 11:**
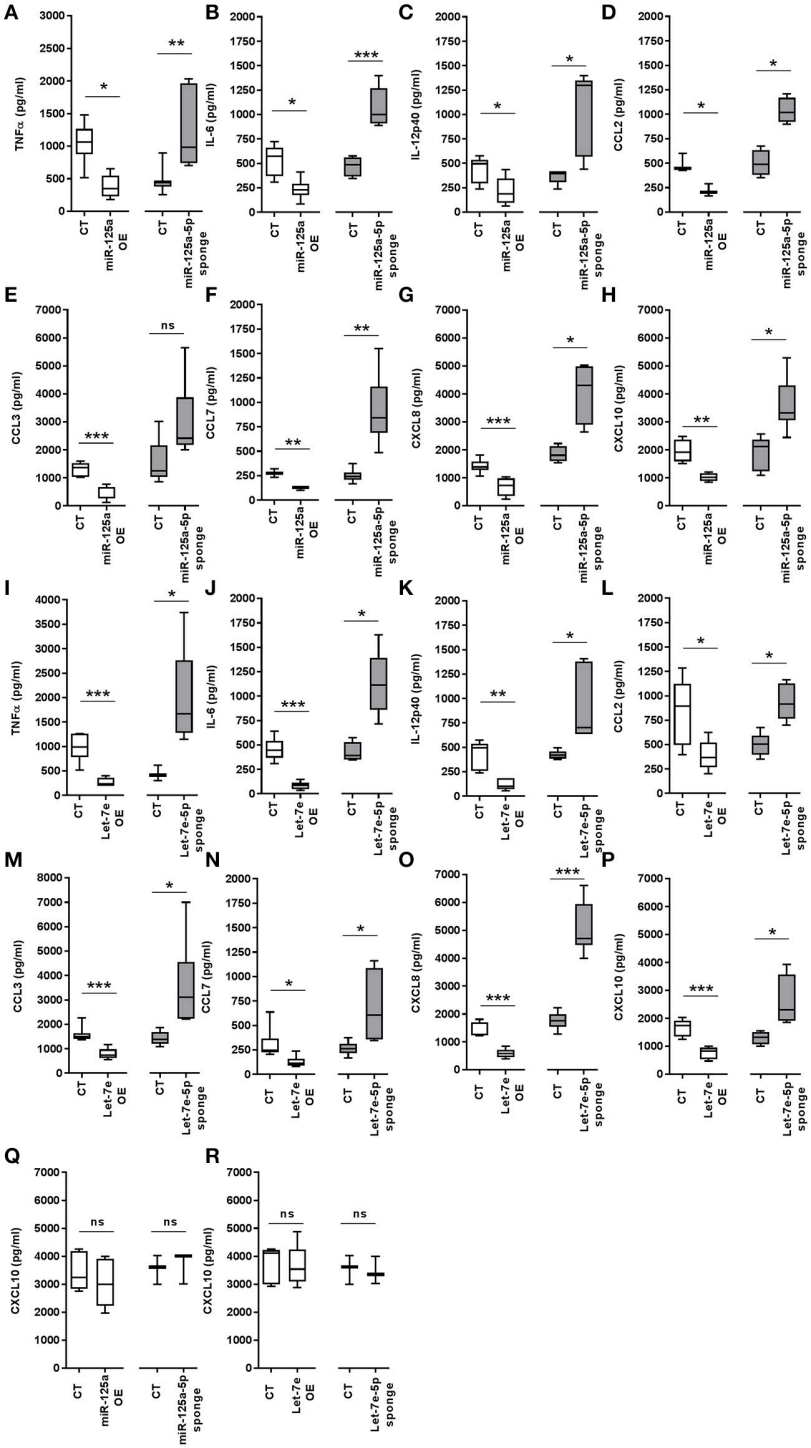
MiR-125a-5p and let-7e-5p down-regulate pro-inflammatory cytokines. **(A–P)** Levels of pro-inflammatory cytokines measured by ELISA in cell-free supernatants of THP-1 cells transduced with CT, miR-125a OE, and let-7e OE, or with miR-125a-5p sponge, let-7e-5p sponge after stimulation with 1 μg/mL LPS for 8 h for TNFα **(A,I)**, CCL3 **(E,M)**, and CXCL8 **(G,O)**, or 24 h for IL-6 **(B,J)**, IL-12p40 **(C,K)**, CCL7 **(F,N)**, CCL2 **(D, L)**, and CXCL10 **(H,P)**. Results of 6 independent experiments are shown. **(Q,R)** CXCL10 levels measured by ELISA in cell-free supernatants of THP1 cells transduced with CT, miR-125a OE, and let-7e OE, or with miR-125a-5p sponge and let-7e-5p sponge, after stimulation with 10 ng/ml IFNγ for 24 h. Results of 5 independent experiments are shown. (* < 0.05; ** < 0.01; *** < 0.001).

As we demonstrated the direct targeting of multiple components of the TLR4 signaling pathway by miR-125a-5p and let-7e-5p, their global impact on TLR4 activity was investigated by evaluating the production of inflammatory cytokines after LPS exposure. As compared to control cells, miR-125a OE THP-1 cells showed a significant reduction in LPS-dependent production of several inflammatory cytokines, including TNFα, IL-6, IL-12p40, CCL2, CCL3, CCL7, CXCL8, and CXCL10, while a significant increase of these pro-inflammatory cytokines was observed when miR-125a-5p was inhibited (miR-125a-5p sponge THP-1 cells; Figures 11A–H). Similar results were obtained when let-7e-5p was modulated using the same approaches (Figures 11I–P). Of note, miR-125a-5p and let-7e-5p showed significant effects on both cytokines identified as direct targets (TNFα, IL-6, CCL3, CCL7, CXCL8; see Figure 10) as well as others not predicted as direct targets (IL-12p40, CCL2, CXCL10; see Figure 6), consistent with a global effect of these miR on the TLR4 signaling pathway. After stimulation with LPS, CXCL10 protein levels were also significantly inhibited in miR-125a OE and let-7e OE THP-1 cells, and increased in miR-125a-5p sponge and let-7e-5p sponge THP-1 cells (Figures 11H,P, respectively), while no effect was observed when this chemokine was induced by IFNγ (Figures 11Q,R), confirming that the effects of miR-125a-5p and let-7e-5p were mediated by their specific targeting of the TLR4 signaling pathway. Taken together, these data indicate that miR-125a-5p and let-7e-5p operate as anti-inflammatory miRs dampening down the proinflammatory activity of LPS by multiple targeting of key components in the TLR4 signaling pathway.

### The miR-125a~99b~let-7e cluster mediates LPS tolerance

**Figure 12:**
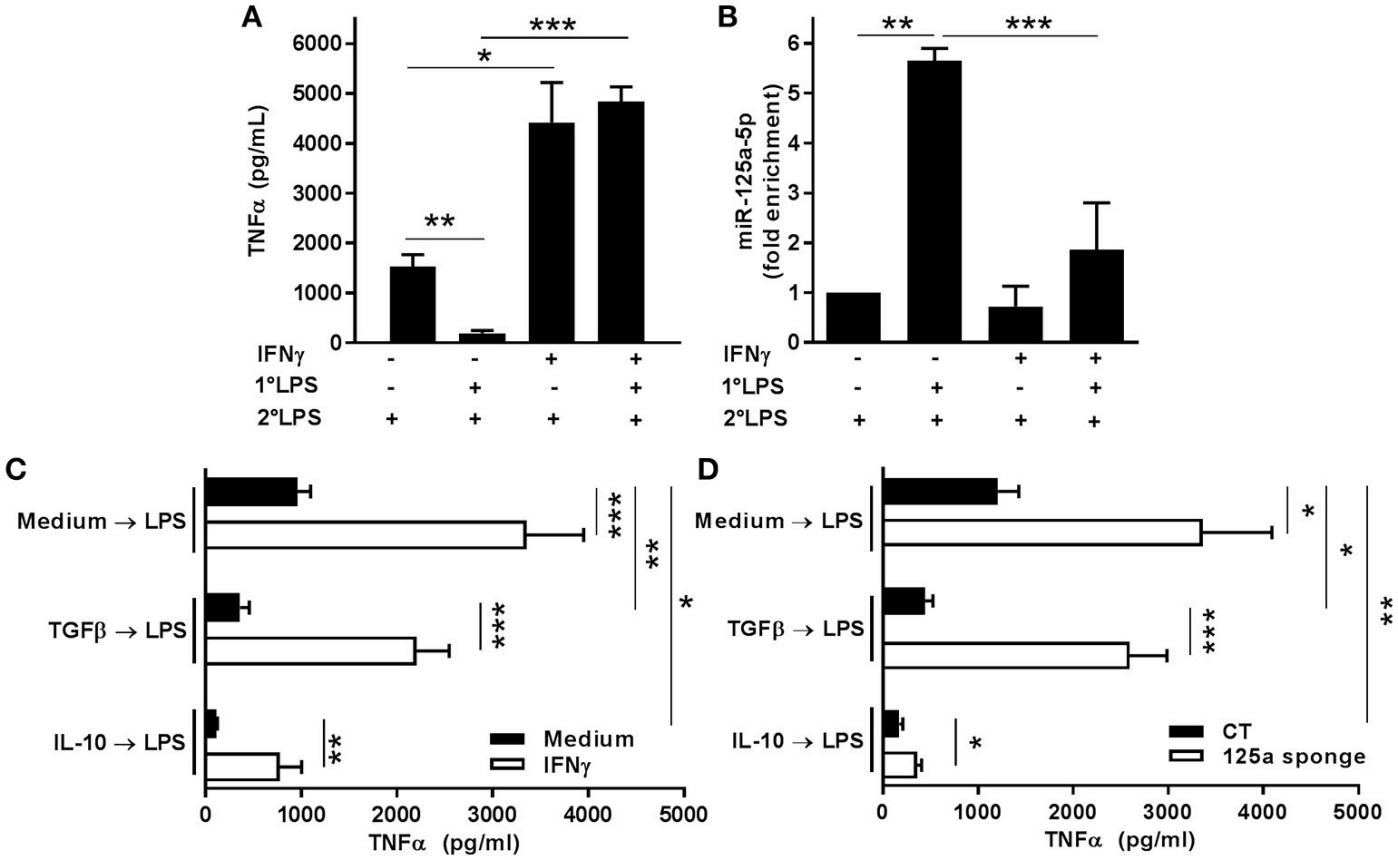
MiR-125a-5p is expressed in tolerized monocytes and contributes to IL-10 and TGFβ-dependent tolerance. **(A,B)** Monocytes were pretreated or not with 10 ng/ml IFNγ, primed or not with 0.1 ng/ml LPS (1°LPS) for 18 h, and then challenged with 10 ng/ml LPS (2°LPS). After 24 h, TNFα levels were measured by ELISA in cell-free supernatants **(A)**, and expression levels of miR-125a-5p was assayed by Q-PCR in triplicate samples **(B)**. Results are expressed as fold change over control (mean ± SEM; *n* = 5). **(C)** Untransfected THP-1 cells incubated or not ON with 10 ng/ml IFNγ (white and black columns, respectively) were pre-treated or not with TGFβ or IL-10 for 2 h before stimulation with 100 ng/ml LPS for 48 h. TNFα levels were measured by ELISA in cell free supernatants. Results are shown as mean ± SEM of 3 independent experiments. **(D)** THP-1 cells transduced with miR-125a-5p sponge or control vectors (white and black columns, respectively) were pre-treated or not with TGFβ or IL-10 for 2 h before stimulation with 100 ng/ml LPS for 48 h. TNFα levels were measured by ELISA in cell free supernatants. Results are shown as mean ± SEM of 5 independent experiments. (* < 0.05; ** < 0.01; *** < 0.001).

As our results demonstrated that the miR-125a~99b~let-7e cluster was an effective negative regulator of TLR4 signaling, we investigated its involvement in the induction of LPS tolerance. As shown in Figures 12A,B, the impaired production of TNFα observed in LPS tolerant monocytes was paralleled by a significant increase in their expression of miR-125a-5p, and the ability of IFNγ to prevent the induction of LPS tolerance, as shown by IFNγ-dependent rescue of TNFα production, correlated with its ability to prevent the accumulation of miR-125a-5p in tolerant monocytes. Consistent with the LPS tolerant behavior previously described in monocytes (1, 30), THP-1 cells exposed to TGFβ or IL-10 also significantly reduced TNFα production after LPS challenge, while IFNγ pre-treatment strongly enhanced LPS-dependent production and significantly impaired the tolerization effect of TGFβ and IL-10 (Figure 12C). In this setting, miR-125a-5p inhibition in THP-1 cells resulted in increased TNFα production as compared to CT cells, and blocking TGFβ- or IL-10-induced miR-125a-5p expression was sufficient to revert their LPS tolerogenic effect (Figure 12D). Taken together, these results indicate that the miR-125a~99b~let-7e cluster is a molecular effector of IL-10- and TGFβ-dependent pathways negatively regulating the LPS inflammatory signal and that miR-125a-5p acts as an intracellular mediator of LPS tolerance.

## Discussion

Excessive inflammation can be detrimental to the host and, consequently, several regulatory mechanisms have evolved to control its magnitude and duration (31–33). IL-10 and TGFβ are key feedback inhibitors of the TLR signaling pathway primarily acting at both transcriptional (26) and post-transcriptional levels (27, 34) to inhibit pro-inflammatory cytokines production. To investigate the potential role of miRs in the post-transcriptional mechanisms dampening innate immune cell activation, we extensively characterized the expression of the evolutionary conserved LPS-responsive miR-125a~99b~let-7e cluster in response to different pro- and anti-inflammatory signals, including TLR ligands and pro-and anti-inflammatory cytokines. Our results indicate that the miR-125a~99b~let-7e cluster acts as a gene late-induced by LPS in monocytes as the effect of an IL-10-dependent regulatory loop and is counter-regulated by IFNγ, which promotes macrophage classic pro-inflammatory activation and chronic inflammation (4) (Figure S2). This expression pattern is consistent with previous reports showing that expression of both let-7e-5p and miR-125a-5p is associated with the development of an appropriate host defense. In particular, let-7e over-expression led to the rescue of defect in LPS tolerance in AKT-1^−−/−−^ mice (18), and miR-125a-5p has been proposed to suppress classical proinflammatory activation and promote the anti-inflammatory alternative activation of macrophages (35).

The analysis of the core promoter region of miR-125a~99b~let-7e cluster identified different transcriptional binding sites depending upon pro- or anti-inflammatory mediators investigated. The three miRs in the cluster were always coregulated, suggesting they could act coordinately to control a target biological process. To investigate this hypothesis, we evaluated their potential involvement in the coregulation of complex gene regulatory networks adopting a computational method based on GO term enrichment and IPA analysis, which revealed that predicted targets of miR-125a-5p and let-7e-5p were significantly enriched in functional cathegories associated with the inflammatory response and macrophage activation in particular, with multiple key genes being predicted as targets for both miR-125a and let-7e. Computational methods, complemented by experimental target validation, made possible the reconstruction of large scale biological gene networks associated with the list of miR-125a~99b~let-7e cluster predicted targets. Interestingly, the main gene regulatory network generated by predicted target genes of miR-125a-5p and let-7e-5p was an “inflammatory network” significantly enriched of nodes and edges centered on the TLR pathway. Notably, miR-99b-5p, which has an overall significantly lower number of predicted targets when compared to miR-125a-5p and let-7e-5p (1171 vs. 6148 and 4891 target genes, respectively), was not predicted to be actively involved in the modulation of the TLR pathway. The observation that also miR-99b-5p target genes are enriched in the genonthology category *Immune system* (Figure 5A) but in different functional subcategories as compared to the target genes of the other two miR (Figure 5B) suggests the association of this miR with different aspects of the immune response. In particular, miR-99b-5p not only is not involved in the regulation of the TLR pathway, but target gene enrichment analysis also does not support evidence for its role in macrophage activation (Figure 5B). On the contrary, this miR, and not miR-125a-5p and let-7e-5p, is significantly enriched for genes associated with the subcategory *Cellular growth and profileration* part of the *Immune system* genonthology category GO term 0002376 (Figure 5B), and it is tempting to speculate it might be involved in controlling the emeriging potential of macrophage to profilerate (36). Collectively, these results indicated that miR-125a-5p and let-7e-5p cooperatively regulate key genes of the TLR4 signaling pathway, possibly acting in concert through the subtle individual regulation of multiple genes rather than operating a strong repression of isolated targets, a multistep approach previously described in other biological settings (18, 37). This pervasive regulation involved targeting of common components at multiple levels of the TLR signaling pathway, including receptors (e.g., TLR4 and CD14) and signal transducers (e.g., IRAK1), with the resulting effect of a global suppression of downstream inflammatory cytokine levels. This biological function is consistent with the induction of miR-125a~99b~let-7e cluster by agonists activating different TLR/IL-1R pathways and highlights the importance of multiple checkpoints in the signaling cascade to allow the controlled development of an appropriate inflammatory response. We also demonstrated the induction of miR-125a~99b~let-7e cluster expression by IL-10 and TGFβ and showed evidence that this induction is biologically relevant, as miR-125a-5p mediated IL-10- and TGFβ-dependent tolerance to LPS. In this setting, our evidence showing the negative regulation of this miR cluster by IFNγ and the ability of miR-125a-5p inhibition to reverse the tolerance state point to the down-regulation of this miR cluster as one of the mechanisms by which IFNγ induce a state of refractoriness to the suppressive effects of TGFβ and IL-10 (Figure S2). Consistent with this, we showed that manipulation of this miR cluster significantly shapes the profile of inflammatory cytokines induced downstream by TLR agonists.

In conclusion, our findings identified the miR-125a~99b~let-7e cluster as a new key intracellular mediator used by the master anti-inflammatory cytokine IL-10 to dampen down the pro-inflammatory TLR pathway and provides a new perspective in the understanding of mechanisms by which the inflammatory response is regulated in phagocytes. Further studies are required to fully disclose the role of miR-125a-5p and let-7e-5p in the resolution of inflammation and in endotoxin tolerance and to evaluate the potential of their exploitation to develop novel therapeutic approaches for inflammatory diseases.

## Author contributions

GC designed and performed most of the experiments and analyzed data. GC, MA, MD, and MM generated the luciferase-reporter constructs and performed the luciferase assays. TR, MA and GC performed and analyzed ChIP data. TR and MR performed RIP assays. GC performed bioinformatics analysis. LD contributed to manuscript preparation and revision. FB discussed the results and implications and commented on the manuscript. ML designed and supervised the study, and wrote the paper.

### Conflict of interest statement

The authors declare that the research was conducted in the absence of any commercial or financial relationships that could be construed as a potential conflict of interest.
