# Supplementary material for: Multi-Step Regulation of the TLR4 Pathway by the miR-125a~99b~let-7e Cluster
Source: Front Immunol. 2018 Sep 7;9:2037. doi: 10.3389/fimmu.2018.02037 (PMC6137199; doi:10.3389/fimmu.2018.02037)
Supplement: Supplementary file 1 [file Table_1.DOC]

**SUPPLEMENTAL MATERIAL**

**Table S1. List of oligonucleotides used.**

| **Cloning** | TLR4-3’UTR | 5’-AATACAGAGTCTTCCAGGTG-3’  5’-TGTTCAATCACCCTAGACCT-3’ |
| --- | --- | --- |
|  | CD14-3’UTR | 5’-TGGATAACCTGACACTGGA-3’  5’-ATGAAGAAAGCCTAAGTATG-3’ |
|  | IRAK1-3’UTR | 5’-ATCATTTATGCTTGGGAGGT-3’  5’-AAGAGGACACTCGGTTACA-3’ |
|  | IL-6-3’UTR | 5’-GTCAGAAACCTGTCCACT-3’  5’-AATATGTATAAGTTAGCCAT-3’ |
|  | CXCL8-3’UTR | 5’-CCAAGAGAATATCCGAACT-3’  5’- CAAAGAGAATCCCAATAAGC-3’ |
|  | TNFα-3’UTR | 5’-GGAGGACGAACATCCAACCT-3’  5’-AGCAATGAGTGACAGTTGGTCA-3’ |
|  | CCL3-3’UTR | 5’-CTGAGCCTTGGGAACAT-3’  5’-AGAGCATCTTTATTATTTCC-3’ |
|  | CCL7-3’UTR | 5’-ACATTCATGACTGAACTGA-3’  5’-ACAAAAATCTATTTTAT-3’ |
|  | miR-125a | 5’-TGCCTATCTCCATCTCTGACC-3’  5’-TGGTGGTCAAATGTCATGCT-3’ |
|  | let-7e | 5’-CTGTCTGTCTGTCGGGTCTG-3’  5’-GCAGGGACAAGGACAGAAAA-3’ |
| **Seed mutagenesis** | miR125a-5p seed  in TLR4-3’UTR | 5’-AAGGACAATCAGGATGTCATAAATGAAAATAAAAACCACAATG-3’  5’- CATTGTGGTTTTTATTTTCATTTATGACATCCTGATTGTCCTT-3’ |
|  | let-7e-5p seed  in TLR4-3’UTR | 5’- CCATGACAAAGAAAGTCATTTCAACTCTTATCAAGTTGAATAA-3’  5’-TTATTCAACTTGATAAGAGTTGAAATGACTTTCTTTGTCATGG-3’ |
|  | miR125a-5p seed  in CD14-3’UTR | 5’-CTGCCTTGGCTTCGAGTCCCGTCAGG-3’  5’-CCTGACGGGACTCGAAGCCAAGGCAG-3’ |
|  | miR125a-5p seed 1  in IRAK1-3’UTR | 5’-CAGACAGGGAAGGGAAACATTTTGAAAAGACATGTATCAC-3’  5’-GTGATACATGTCTTTTCAAAATGTTTCCCTTCCCTGTCTG-3’ |
|  | miR125a-5p seed 2  in IRAK1-3’UTR | 5’-CAGGGAAGGGAAACATTTTGGACATGTATCACATGTCTTC-3’  5’-GAAGACATGTGATACATGTCCAAAATGTTTCCCTTCCCTG-3’ |
|  | miR125a-5p seed  in TNFα-3’UTR | 5’-TCTGGAATCTGGAGACAGCCTTTGGTTCTGGC-3’  5’-GCCAGAACCAAAGGCTGTCTCCAGATTCCAGA-3’ |
|  | let-7e-5p seed  in IL-6-3’UTR | 5’-GTCAGAAACCTGTCCACT-3’  5’-AATATGTATAAGTTAGCCAT-3’ |
|  | miR125a-5p seed  in CCL3-3’UTR | 5’-AAATGTGTATCGGATGCTTTTGTGGCTGTGATCGG-3’  5’-CCGATCACAGCCACAAAAGCATCCGATACACATTT-3’ |
|  | let-7e-5p seed  in CCL3-3’UTR | 5’-GTGTGACCTCCACAGCTTTCTATGGACTGGTTGT-3’  5’-ACAACCAGTCCATAGAAAGCTGTGGAGGTCACAC-3’ |
|  | miR125a-5p seed  in CXCL8-3’UTR | 5’-GATGTTTTATTAGATAAATTTCGGGTTTTTAGATTAAAC-3’  5’-GTTTAATCTAAAAACCCGAAATTTATCTAATAAAACATC-3’ |
|  | let-7e-5p seed  in CXCL8-3’UTR | 5’-AAGTATTAGCCACCATCTCACAGTGATGTTGTGAGG-3’  5’-CCTCACAACATCACTGTGAGATGGTGGCTAATACTT-3’ |
|  | let-7e-5p seed  in CCL7-3’UTR | 5’-ATGCTCCTCCCTTCTCCATGGGGGTATTGTA-3’  5’-TACAATACCCCCATGGAGAAGGGAGGAGCAT-3’ |
| **Q-PCR** | TLR4 | 5’-CACCTGATGCTTCTTGCTG-3’  5’-TCCTGGCTTGAGTAGATAA-3’ |
|  | CD14 | 5’-GCAACACAGGAATGGAGA-3’  5’-ACAGATTGAGGGAGTTCA-3’ |
|  | IRAK1 | 5’-TGAAGAGGCTGAAGGAGAA-3’  5’-CACAATGTTTGGGTGACGAA-3’ |
|  | IL-6 | 5’-TACCCCCAGGAGAAGATTCC-3’  5’-TTTTCTGCCAGTGCCTCTTT-3’ |
|  | CCL3 | 5’-TGACTACTTTGAGACGAGCA-3’  5’-CTGACATATTTCTGGACCC-3’ |
|  | CCL7 | 5’-CTGCTGCTACAGATTTATCA-3’  5’-TCCTTGTCCAGTTTGGTCTT-3’ |
|  | CXCL8 | 5’-GCCAGGAAGAAACCACCGGAAGGA-3’  5’-GGGTCCAGACAGAGCTCTCTTCC-3’ |
|  | TNFα | 5’-GCTGCACTTTGGAGTGATCG-3’  5’-GAGGTACAGGCCCTCTGATG-3’ |
|  | GAPDH | 5’-GATCATCAGCAATGCCTCCT-3’  5’-TGTGGTCATGAGTCCTTCCA-3’ |
| **ChIP assay** | Pol II site  (-370 bp from TSS) | 5’-AGGGAAGGGGGAAGAGA-3’  5’-GTGGGGGTGGTTTGAGAA-3’ |
|  | STAT3 site  (-700 bp from TSS) | 5’-AACGCCTTGTCCAGTGACCTT-3’  5’-GTGGGGGTGGTTTGAGAA-3’ |
|  | SMAD3 site  (-1450 bp from TSS) | 5’-GGCAGCGGAAGGGTTAA-3’  5’-CTCCTCCAGACATCTCCT-3’ |
|  | NF-kB site  (-856 bp from TSS) | 5’-TCCCCACCTCCTCTTTAG-3’  5’-GCCCATAGCCCCGCTTTG-3’ |
|  | SP1 site 1  (-320 bp from TSS) | 5’-AGGGAAGGGGGAAGAGA-3’  5’-GTGGGGGTGGTTTGAGAA-3’ |
|  | SP1 site 2  (-420 bp from TSS) | 5’-TTCTCGGCTTCCCCTCT-3’  5’-CAACCTCCCAGACCCTCAG-3’ |
|  | SP1 site 3  (-870 bp from TSS) | 5’-TCCCCACCTCCTCTTTAG-3’  5’-GCCCATAGCCCCGCTTTG-3’ |
|  | SP1 site 4  (-1500 bp from TSS) | 5’-CTGGATTCAAGAGGGAGG-3’  5’-GATTCTGGAATGGGTGGG-3’ |
| **RIP assay** | TLR4 | 5’-CCTCCTCAGAAACAGAACAT-3’  5’-TCATAACGGCTACACCATTT-3’ |
|  | CD14 | 5’-CTAACTCCCTAAGAAACCC-3’  5’-ACCTTTTAATCCAGATGCCA-3’ |
|  | IRAK1 | 5’-CTCTTTGCCCATCTCTTTG-3’  5’-GCCACACTTTTCCAAATTGT-3’ |
|  | MYD88 | 5’-GCTTGGGCTGCTTTTCATT-3’  5’-CCTGCTCACATCATTACAGT-3’ |
|  | TNFα | 5’-CTGACATCTGGAATCTGGA-3’  5’-TCTGGAAACATCTGGAGAG-3’ |
|  | IL-6 | 5’-GCATTCCTTCTTCTGGTCA-3’  5’-ATAGTGTCCTAACGCTCATA-3’ |
|  | CXCL8 | 5’-TACTCCCAGTCTTGTCATTG-3’  5’-TTCCGTAATTCAACACAGCA-3’ |
|  | CCL3 | 5’-AAGCCACCAGACTGACAAA-3’  5’-CCTTTTAAAAGAGCATCTTT-3’ |
|  | CCL7 | 5’-GGATTTTGGTGGGTTTTGAA-3’  5’-TGAGGTAGAGAAGGGAGG-3’ |


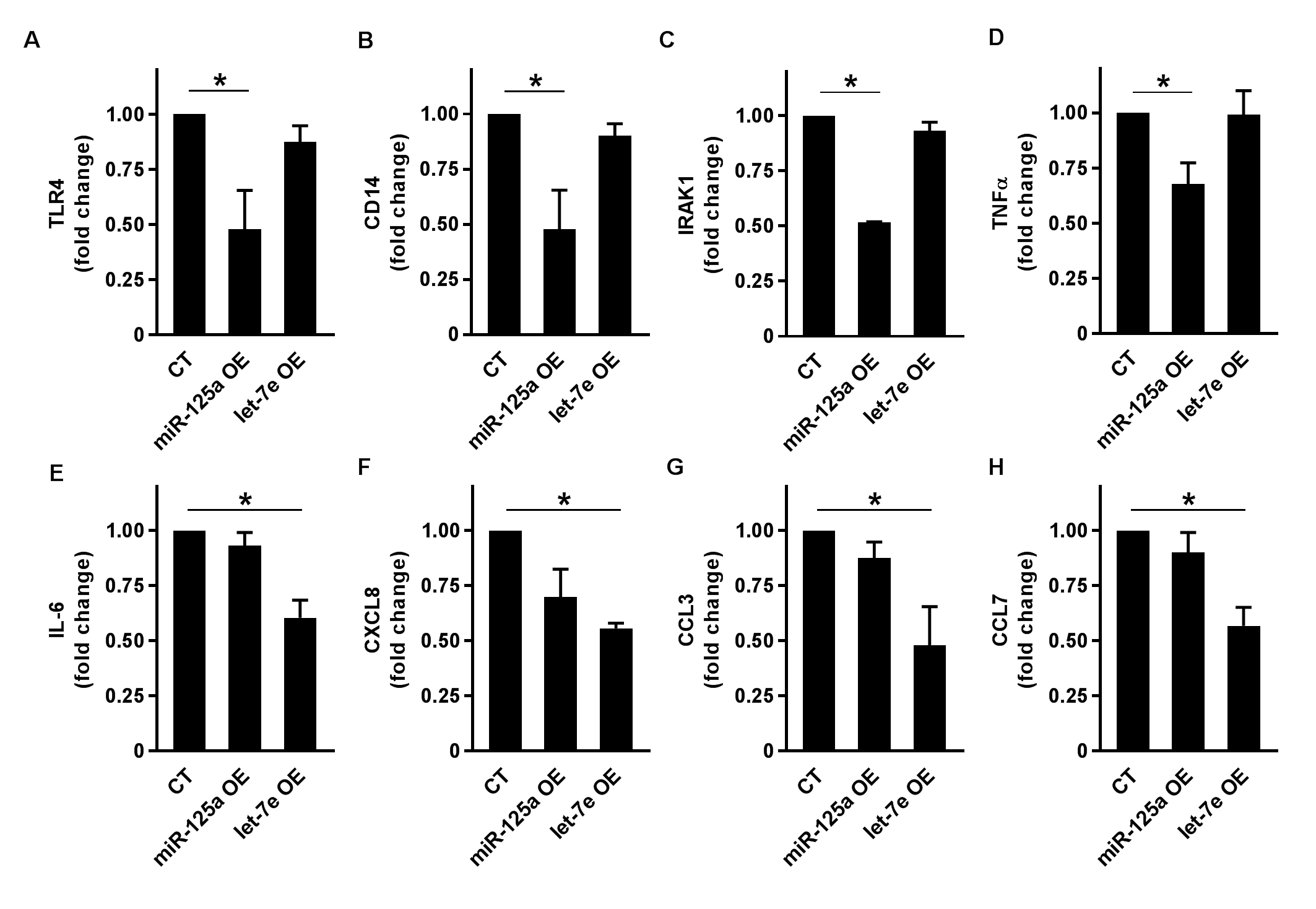


**Figure S1. Effects of miR-125a-5p and let-7e-5p on transcript levels of their direct target genes.**

Levels of mRNA transcripts encoding TLR4 (A), CD14 (B), IRAK1 (C), TNFα (D), IL-6 (E), CXCL8 (F), CCL3 (G), and CCL7 (H) were measured by Q-PCR in CT, miR-125a OE, and let7e OE THP-1 cells stimulated for 4 h with 100 ng/mL LPS and normalized to GAPDH. Results are shown as fold change over non stimulated control (mean ± SEM; n = 3).

**Figure S2. The miR-125a~99b~let-7e cluster is an integrative element of the feedback loops regulating inflammatory networks.**

The TLR4 signaling pathway triggers the induction of pro-inflammatory cytokines, acting in synergism with IFNγ. In the late phase of the inflammatory response, the induction of anti-inflammatory genes, including IL-10 and TGFβ, drives the expression of the miR-125a~99b~let-7e cluster, which negatively modulate the TLR pathway at different steps, thus inducing tolerance. IFNγ interferes with the induction of the tolerant state by inhibiting the induction of anti-inflammatory cytokines and suppressing the expression of the miR-125a~99b~let-7e cluster. Arrowhead lines represent positive regulation and roundhead lines represent negative regulation. Dashed lines report effects of miR-125a~99b~let-7e.
